# Enhanced Activation of Peroxymonosulfate for Tetracycline Degradation Using CoNi-Based Electrodeposited Films

**DOI:** 10.3390/nano13050790

**Published:** 2023-02-21

**Authors:** Elvira Gómez, Arnau Fons, Roberto Cestaro, Albert Serrà

**Affiliations:** 1 Grup d’Electrodeposició de Capes Primes i Nanoestructures (GE-CPN), Departament de Ciència de Materials i Química Física, Universitat de Barcelona, Martí i Franquès, 1, 08028 Barcelona, Catalonia, Spain; 2 Institute of Nanoscience and Nanotechnology (IN2UB), Universitat de Barcelona, 08028 Barcelona, Catalonia, Spain; 3 Empa Swiss Federal Laboratories for Materials Science and Technology, Laboratory for Joining Technologies and Corrosion, CH-8600 Dübendorf, Switzerland

**Keywords:** CoNi, electrodeposition, PMS, sulfate radicals, heterogeneous catalysis, tetracycline, water treatment

## Abstract

Synthesizing efficient heterogeneous catalysts with multiple active sites able to activate peroxymonosulfate (PMS) for the degradation of persistent organic pollutants continues to be a challenge for societies worldwide. In response, cost-effective, eco-friendly oxidized Ni-rich and Co-rich CoNi micro-nanostructured films were fabricated following a two-step process based on simple electrodeposition using green deep eutectic solvent as an electrochemical media and thermal annealing. The CoNi-based catalysts demonstrated exceptional efficiency in the heterogeneous catalyzed activation of PMS for tetracycline degradation and mineralization. The effects of the catalysts’ chemical nature and morphology, the pH, the concentration of PMS, irradiation with visible light, and the duration of contact with the catalysts on the degradation and mineralization of tetracycline were also studied. In dark conditions, oxidized Co-rich CoNi degraded more than 99% of tetracyclines in only 30 min and mineralized more than 99% of them in only 60 min. Moreover, the degradation kinetics doubled from 0.173 min^−1^ in dark conditions to 0.388 min^−1^ under visible light irradiation. In addition, the material demonstrated excellent reusability and can be easily recovered with simple heat treatment. Given those findings, our work provides new strategies for constructing high-efficiency and cost-effective PMS catalysts and elucidating the effects of operational parameters and primary reactive species formed by the catalyst–PMS system on water treatment technologies.

## 1. Introduction

Water, although covering over 70% of Earth’s surface, is a scarce resource, with only 2.5% of it being fresh and most of it being inaccessible (e.g., glaciers, polar ice caps, and underground aquifers). As the world’s population grows, urbanization and economic development continue to drive up the demand for water, leading to a critical water crisis [1]. According to the World Health Organization, over 2 billion people lack access to safe drinking water. Water pollution also presents a significant problem, with an estimated 80% of global wastewater discharged back into the environment without adequate treatment, contaminating rivers, lakes, and groundwater sources. The water crisis has severe consequences for global development, health, and poverty eradication. Insufficient access to clean water affects hygiene and sanitation, leading to the spread of diseases such as cholera, typhoid, and diarrhea. Moreover, the crisis has the potential to cause socioeconomic instability, ecological degradation, and conflict. It is, therefore, critical to ensure that access to water is not only viewed as an essential resource for ecosystem maintenance and economic development but as a basic human right that needs to be observed to ensure the life and dignity of human beings worldwide [2].

In developed countries, water purification systems allow access to clean water and return water to the environment in good condition through physical, chemical, and biological processes. Nevertheless, those techniques cannot remove many contaminants from wastewater because treatment plants lack the capacity to effectively remove various emerging pollutants. Despite several procedures for eliminating persistent contaminants from aquatic media—membrane filtration, adsorption, photocatalytic degradation, and electrochemical oxidation, to name a few, some of which are labeled advanced oxidation processes (AOPs) [2,3,4]—they are not all universal, applicable worldwide, or capable of completely eliminating all persistent and emerging pollutants. To counter that trend, new methods of efficiently eliminating persistent contaminants present in water and achieving complete water purification need to be developed and assessed [2,5,6].

Recent studies have highlighted the potential of sulfate radicals for mineralization, as they can partially or completely oxidize organic contaminants [7,8]. The standard reduction potential of sulfate radicals (2.5–3.1 V) is exceptionally high, which gives them a high degree of oxidizing power in organic decomposition. Activation of peroxymonosulfate (PMS) or persulfate (PS) can produce sulfate radicals through various mechanisms, including homolytic cleavage of the peroxide bond, electron transfer, and radical coupling reactions [7,8]. The radicals formed by the activation of PMS degrade the contaminant by mineralizing it entirely and thereby yielding CO_2_, H_2_O, and other inorganic salts as final products. In effect, that dynamic precludes the risk of the formation of secondary species that could be even more harmful [9].

Peroxomonosulfate is an environmentally friendly oxidant that can be activated by various methods including ultrasound technology, microwave irradiation, ultraviolet light, and electrolysis, although the most applicable, especially in potential large-scale processes, is activation via interaction with catalytic surfaces [10]. Traditional transition metal oxides may be efficient catalysts for activating PMS, the activation of which by transition metals has numerous benefits, including ease of use under ambient conditions [11]. Among all transition metal ions, Co^2+^ is the most efficient for activating PMS. However, the high biotoxicity of Co (II) introduced via catalyst leaching during the degradation process has been shown to limit the development of Co(II)-based PMS catalysts [12,13,14,15]. Considering that mixed catalysts with more than one metal usually present additional advantages compared with single metal oxides, including greater stability and activity [16], the development of various bimetallic Co-based PMS catalysts has aroused interest among researchers. Of all Co-based bimetallic candidates, CoNi-based catalysts have demonstrated exceptional efficiency in activating PMS due to relatively high electronic stability that provides multiple-redox active sites as well as reduces catalyst leaching [17]. The activation of PMS with transition metals to generate sulfate radicals involves charge transfer and an active reversible redox cycle of Co^2+^–Co^3+^ and Ni^2+^–Ni^3+^, as shown in previous studies [18,19,20]:

(1) $$M{\left(OH\right)}^{\left(n-1\right)+}+HSO_{5}^{-}\to M{\left(OH\right)}^{n+}+OH^{-}+SO_{4}^{●-}$$

(2) $$M{\left(OH\right)}^{n+}+HSO_{5}^{-}\to M{\left(OH\right)}^{\left(n-1\right)+}+H^{+}+SO_{5}^{●-}$$

In the work presented here, electrodeposited cobalt and nickel alloys (CoNi) were used as activator precursors. Two different metal proportions were selected to evaluate their effectiveness in activating PMS given that the synergetic effects of both metals could complement CoNi as a good candidate for PMS catalysis [20]. In a previous study addressing the electrochemical preparation of CoNi alloys from a sustainable deep eutectic solvent (DES), as a proof of concept, some alloy compositions have been used as catalysts for activating PMS and showed highly promising results [21]. For a contaminant model, the antibiotic tetracycline (TC) was selected. An emerging contaminant in aquatic media, TC represents a primary group of antibiotics used for veterinary purposes, and, after medication, more than 70% of TC is excreted and released in active form into the environment via urine and feces [22,23,24,25,26,27]. The degradation and mineralization of TC have been studied using UV-Visible spectroscopy and total organic carbon (TOC). The study presented here addressed the influence of different parameters that may modify the efficiency of activating synthesized CoNi, including its composition or subsequent heat treatments. Regarding the solution, the effects of media with pH values close to those of ambient water and the concentration of PMS were evaluated. The effect of visible light and the contact time between the activator and the solutions was also assessed, while chemical stability and catalyst leaching were analyzed to further confirm the potential of CoNi-based PMS catalysts. At all stages, the efficiency and feasibility of reusing the catalyst after several work cycles were considered. The study of the mineralization process was also accompanied by an exhaustive morphological, compositional, and structural characterization of the substrates before and after mineralization.

## 2. Materials and Methods

### 2.1. Materials

All chemicals used in the study were of analytical grade and used without further purification. The purity of the chemicals is summarized as follows: choline chloride (Across Organics, Geel, Belgium 99%), urea (Merck, Darmstadt, Germany 99%), cobalt (II) chloride hexahydrate (Alfa Aesar, Karlsruhe, Germany 99%), nickel (II) chloride hexahydrate (Merck, Darmstadt, Germany 99%), hydrochloric acid (Merck, Darmstadt, Germany 37%), sodium hydroxide (Merck, Darmstadt, Germany >98%), peroxymonosulfate (Sigma-Aldrich, Gillingham, UK), tetracycline (Alfa Aesar, Karlsruhe, Germany 96%), methanol (Merck, Darmstadt, Germany >99.8%), and tert-butyl alcohol (Merck, Darmstadt, Germany >99.5%). All aqueous solutions were prepared using MilliQ-treated water.

### 2.2. Synthesis of PMS-Catalysts

Following published protocols, metallic CoNi surfaces were fabricated by electrodeposition in a deep eutectic solvent (DES) solution prepared by mixing choline chloride (Across Organics, Geel, Belgium 99%) and urea (Merck, Darmstadt, Germany 99%) in a 1:2 molar ratio at 50 °C under constant stirring. Cobalt (II) chloride and nickel (II) chloride hexahydrate of 99% purity were purchased from Alfa Aesar and Merck, respectively [21,28,29]. Prior to preparing the electroactive solutions, the salts were dried at 105 °C for 24 h until dehydration. Two solutions with different [Co(II)]/[Ni(II)] molar ratios were used.

Electrochemical experiments were performed in a thermostated three-electrode system using Ag|AgCl as the reference electrode and a platinum spiral as the counter-electrode. The substrates acting as working electrodes were flat silicon pieces with an area of 0.25 cm^2^ and a seed layer containing Ti (i.e., 15 nm) and Au (i.e., 100 nm). Potentiostatic electrodeposition was performed with an Autolab using PGSTAT30 equipment and NOVA 2.0 software at different deposition potentials to obtain Ni- and Co-rich CoNi micro-nanostructured films. After preparation, the samples were cleaned with warm water to remove the residues of the solvent.

To promote oxide formation in each alloy composition, some samples were subjected to two different heat treatments, both for 2 h in the oven in an air atmosphere, following the protocol described in Figure S1.

### 2.3. Characterization of PMS Catalysts

The morphology of the samples before and after use as catalytic platforms was imaged using field emissions scanning electron microscopy (FE-SEM; Jeol, Bruxelles, Europe, JSM-7100F Analytical Microscope), and their compositions were characterized using coupled energy dispersive spectroscopy (EDS) at 20 kV following the calibration of the equipment using a pure cobalt standard. Elemental composition was also confirmed by inductively coupled plasma mass spectrometry (ICP-MS; Perkin-Elmer spectrometer, Waltham, MA, USA, Elan 6000). Films were dissolved in 1% nitric acid, and the resulting solutions were analyzed by ICP-MS to confirm the deposit composition.

Similarly, before and after the use of all samples, their characterization with X-ray photoelectron spectroscopy (XPS) and X-ray diffraction (XRD) was performed, and XRD patterns were recorded using a Bruker D8 Discovery diffractometer (Karlsruhe, Germany) in the Bragg–Brentano configuration and Cu K-α radiation (λ = 0.1542 nm). A 2-θ scan between 20° and 90° was used with a step size of 0.05 and a measurement interval of 15 s per step. All spectra were collected using Al K_α_ radiation at 1486.6 eV. XPS measurements of the samples were made on a representative circle 0.8 mm in diameter in an ultra-high vacuum chamber with an XPS system (PHI 5600 Multitechnique, Physical Electronics) using a monochromatic X-ray source (Al K_α_line = 1486.6 eV, 350 W). The compositional profile of the CoNi deposits was obtained after measuring each of the successive etch cycles with argon ion sputtering down to a few nanometers. MultiPak 8.2 was used to acquire digital images and perform peak deconvolution.

### 2.4. Catalytic Experiments

The ability of the different deposits prepared for activating PMS (oxone), which contains KHSO_5_·0.5KHSO_4_·0.5K_2_SO_4_ (Merck, Darmstadt, Germany), to generate oxidative radical species was evaluated by studying the degradation and mineralization of tetracycline (TC, Alfa Aesar, Ward Hill, MA, USA). TC solutions were prepared in solutions with different pH levels using the convenient addition of hydrochloric acid (HCl) or sodium hydroxide (NaOH). The degradation of TCs was studied by measuring absorbance as a function of time with a spectrophotometer (UV-1800 Shimadzu). To study the kinetics of degradation, we filled a quartz cuvette with 2.5 mL of the TC solution (i.e., 20 ppm) and left the sample for 30 min to allow its adsorption. Afterward, 0.1 mL of PMS solution was added to the cuvette, such that the concentration of PMS attained the proposed value, at which point we began monitoring the absorbance in the reaction medium. The concentration of TC in both the fresh and catalyzed solutions was analyzed using liquid chromatography-mass spectroscopy (LC-MS) on a Waters Xevo G2-XS QToF system. Mineralization was evaluated by determining the total organic content (TOC) in the solution (TOC-VCSH, Shimadzu, Kyoto, Japan). Free radical quenching experiments were performed using methanol (MeOH) and tert-butyl alcohol (TBA) as scavengers to investigate the role of reactive oxygen species formed during the process. EtOH quenches hydroxyl and sulfate radicals, while TBA quenches only hydroxyl radicals. By comparing the degradation values in the presence of these agents, the effects of both radical species can be distinguished. Last, ICP-MS was used to analyze the cobalt and nickel ions dissolved during degradation and mineralization.

## 3. Results and Discussion

### 3.1. Synthesis and Characterization of PMS-Catalysts

Although Co-based oxide nanomaterials are preferred activators of PMS [19,30,31,32], Cai et al. have demonstrated that the synergistic effect between Ni and Co can magnify the catalytic performance of cobalt oxides by increasing the electron transfer for the enhanced breakage of O–O bonds [33]. To investigate the synergistic effect between Ni and Co on the activation of PMS, Co-rich and Ni-rich CoNi (deposits hereinafter referred to as “Co–CoNi” and “Ni–CoNi,” respectively) films were subjected to an annealing treatment at 225 °C and 350 °C for 2 h, respectively [21]. The Co-CoNi and Ni-CoNi deposits were synthesized via simple potentiostatic electrochemical deposition in a DES under constant magnetic stirring at 70 °C. The applied potentials were set at −0.95 V and −1.05 V (vs. Ag|AgCl) in order to obtain Ni- and Co-rich micro-nanostructured films with inverse proportions, respectively (Table 1).

**Figure 1:**
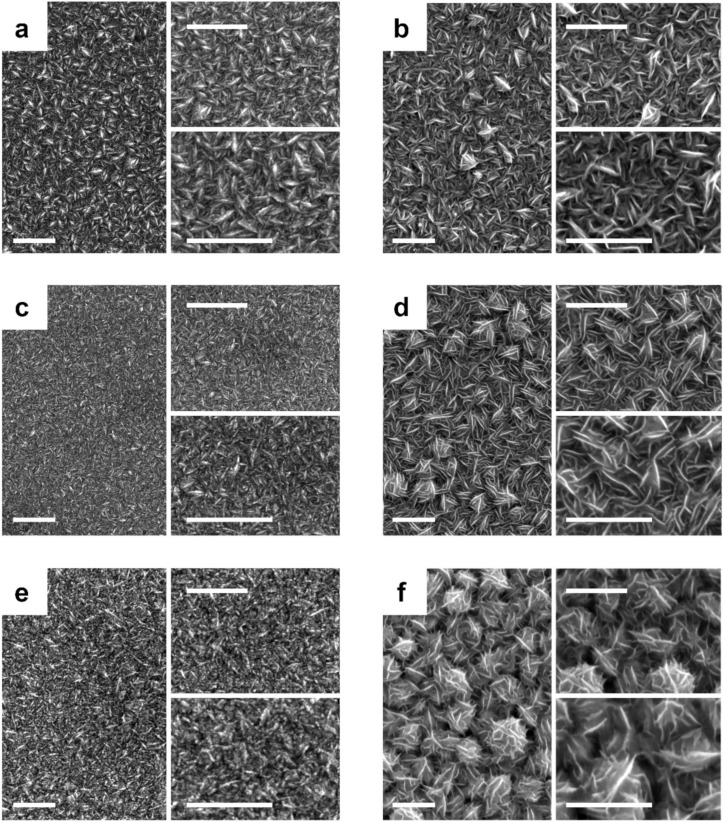
FE-SEM micrographs of (**a**,**c**,**e**) Ni-rich CoNi and (**b**,**d**,**f**) Co-rich CoNi deposits (**a**,**b**) as prepared, (**c**,**d**) after thermal annealing at 225 °C for 2 h, and (**e**,**f**) after thermal annealing treatment at 350 °C for 2 h. Scale bar: 1 µm.

Although the morphology of the deposits depended heavily on the composition of the solution and annealing treatment (Figure 1), all deposits were ultimately compact and homogeneous. Independent of the bath composition, we observed an open morphology involving thin platelets with randomly distributed sharper edges oriented vertically to the surface, thereby appearing to be interwoven (Figure 1a,b). In the case of the Co-rich bath, the compactness was slightly less and the plates sharper. However, in some cases, the grains formed a network-like structure with a highly exposed active area. Added to that, the annealing treatment at 225 °C for 2 h altered the morphology of the deposits. In the case of Ni-CoNi deposits, a rounding of the platelets that blurred the sharp edges and reduced the grain size was observed (Figure 1c). Meanwhile, in the case of Co-CoNi deposits, the platelets were relatively narrow, which enhanced the seemingly interwoven morphology (Figure 1d). After 2 h at 350 °C, the Ni-CoNi deposits were formed by a network with a spongy morphology without sharp corners (Figure 1e), whereas the Co-CoNi deposits presented a desert rose-type morphology, formed by lenticular platelets grouped in the form of petals (Figure 1f). Compared with the Ni-rich deposits, Co-rich deposits presented abundant sharp edges oriented vertically to the surface, which created a large surface area that afforded them more open space and active sites for PMS activation. This kind of architecture may also ensure rapid electron transfer between PMS molecules and active transition metal atoms [21].

**Figure 2:**
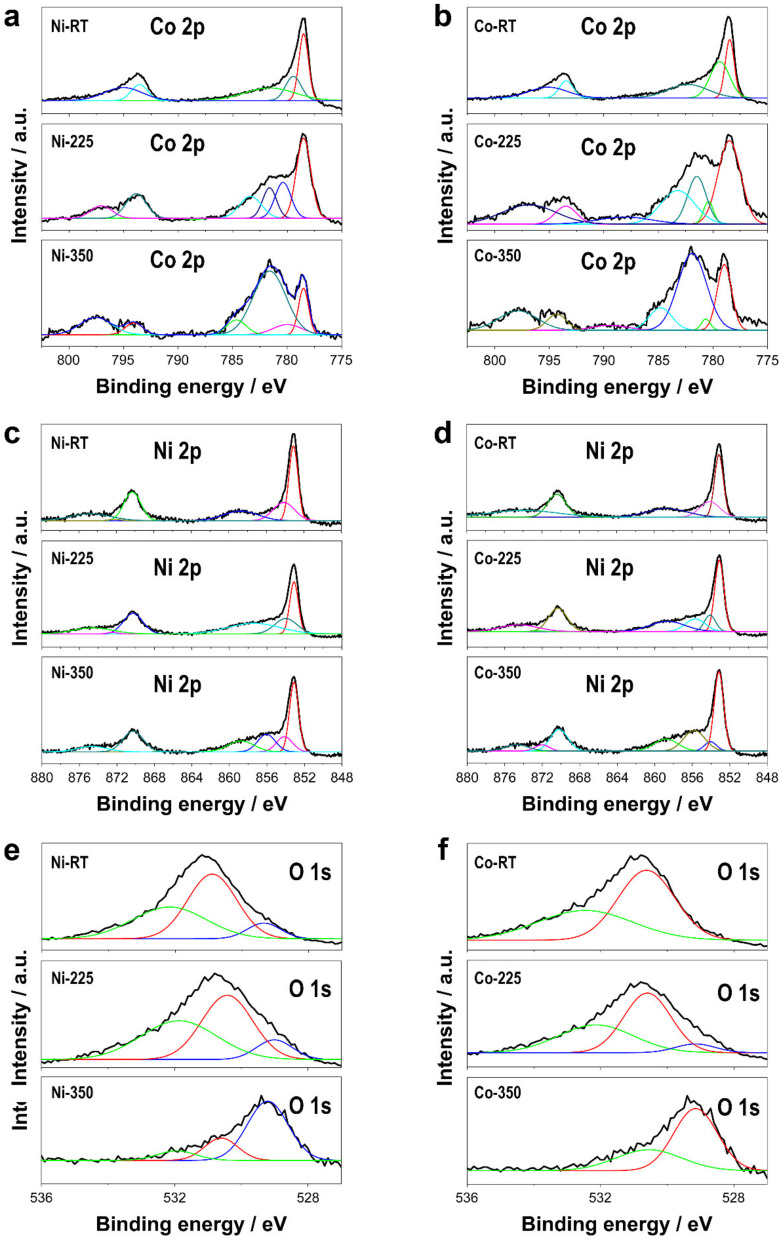
XPS spectra of the deposits as prepared and after thermal treatment of Co 2p of (**a**) Ni–CoNi deposits and (**b**) Co–CoNi deposits, Ni 2p of (**c**) Ni–CoNi deposits, and (**d**) Co–CoNi deposits, and O 1s of (**e**) Ni–CoNi deposits and (**f**) Co–CoNi deposits.

**Figure 3:**
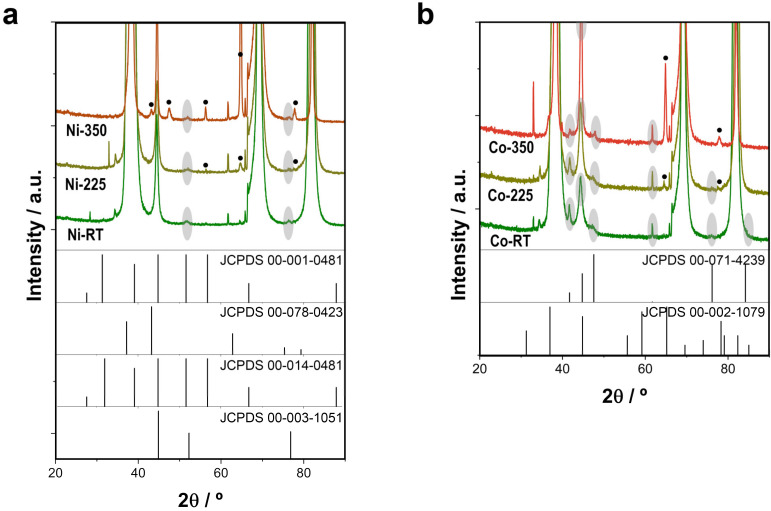
XRD patterns of electrodeposited (**a**) Ni-CoNi and (**b**) Co-CoNi deposits as prepared, after thermal annealing treatment at 225 °C for 2 h, and after thermal annealing treatment at 350 °C for 2 h.

The annealing treatment not only modified the morphology of the deposits but also affected their chemical nature (Figure 2) and crystalline structure (Figure 3). The chemical nature of the Ni, Co, and O of CoNi deposits was characterized by XPS analysis (Figure 2). The binding energies obtained from the analysis were corrected for specimen charging by referencing the C 1s to 284.60 eV. The results revealed that thermal treatment strongly modified the chemical nature of the deposits. The Co 2p spectra of Ni–CoNi and Co–CoNi in particular revealed how the relative amount of Co (II) and Co (III) varied depending on the annealing treatment. At room temperature, the chemical nature of the deposits, regardless of their composition, corresponded primarily to metallic Co, as evidenced by peaks at 778.4 eV and 793.4 eV that are characteristically associated with metallic Co. However, the non-negligible presence of Co^2+^ was also confirmed by peaks at approximately 780.4 eV and 796.2 eV that corresponded to Co^2+^ 2p_3/2_ and Co^2+^ 2p_1/2_ [34,35,36,37]. Thermal treatment in the presence of oxygen promoted the oxidation of the deposit, thereby resulting in the formation of Co (II) and Co (III) oxides. As shown in Figure 2a,b, the asymmetric peaks of Co 2p were also affected by the two spin-orbit doublets—Co 2p_3/2_ (i.e., ~778.4 eV for metallic Co, ~780.1 eV for Co^3+^, and ~781.6 eV for Co^2+^) and Co 2p_1/2_ (i.e., ~794.3 eV for Co^3+^ and ~796.6 eV for Co^2+^)—and two shake-up satellites. The relative amount of Co (II) was significantly higher in deposits treated at 350 °C than at 225 °C, while the relative amount of Co (II) + Co (III) was also higher than that of metallic cobalt. The atom ratio of Co (II) to Co (III), which should have been higher in Co–CoNi samples heat-treated at 350 °C, can translate into higher oxygen vacancy density that can improve the electron transfer between catalysts and PMS, which itself can significantly enhance catalytic performance [34,36,38]. Similarly, the Ni 2p_1/2_ and Ni 2p_3/2_ spectra can be deconvoluted in multiple peaks, which suggests the existence of Ni (0), Ni (II), and/or Ni (III) (Figure 2c,d). The peaks located at approximately 853.1 and 870.3 eV were ascribed to Ni(0)—the binding energies at 854.1 and 872.0 eV corresponded to Ni^2+^, whereas those at 855 eV could be attributed to Ni^3+^—while the peaks at approximately 858.7 and 874.5 eV were two shake-up satellites [37]. Among other results, the formation of Ni^3+^ was evident only after heat treatment at 225 °C and 350 °C in the case of Co–CoNi deposits and only at 350 °C in the case of Ni–CoNi deposits, and deposits with a less Ni content were more easily oxidizable [37,39,40]. Last, the O 1s XPS spectra showed two or three deconvoluted peaks located at binding energies of 531.9, 530.6, and 529.2 eV, which were assigned to physisorbed or chemisorbed water at the surface, adsorbed oxygen, and lattice oxygen at the surface, respectively [34]. The peak located at 529.2 eV, corresponding to a metal–oxygen bond, was more prominent after the annealing treatment than before, which confirms the oxidation of Ni–CoNi and Co–CoNi during the thermal process.

To further confirm the effect of thermal annealing, XRD analysis was performed, which revealed that the most intense diffraction peaks corresponded to the cubic Au and cubic Ti of the Si/Ti/Au substrate. As shown in Figure 3a, the as-prepared Ni–CoNi films had a slightly distorted Ni cubic crystal, with peaks at 44.6°, 51.7°, and 76.3° (JCPDS 00-003-1051) corresponding to a CoNi solid solution. The annealing treatment thus promoted the formation of nickel oxides. Some relevant peaks ascribed to hexagonal Ni_2_O_3_ at 56.4° and 66.1° (JCPDS 00-014-0481) appeared after the annealing treatment, especially at 350 °C. Meanwhile, the peaks at 43.1° and 77.7° can be also ascribed to cubic NiO (JCPDS 00-078-0423), and the peak at 77.7° is compatible with the presence of hexagonal nickel (JCPDS 00-001-0481). The various peaks of hexagonal Ni_2_O_3_, cubic NiO, and hexagonal nickel overlapped with the peaks of cubic nickel [20,21]. By contrast, the as-prepared deposits Co–CoNi films had a slightly distorted Co hexagonal structure (Figure 3b), with peaks at 41.7°, 44.8°, 47.5°, 61.7°, 84.6°, and 76.2° (JCPDS 00-071-4239) also corresponding to a CoNi solid solution. After the thermal annealing treatment, especially at 350 °C, other relevant peaks appeared at 2 θ of 65.0° and 78.3° that can be assigned to cubic Co_3_O_4_ (JCPDS 00-002-1079). Still other peaks of the structure overlapped with Co hexagonal peaks [21]. The formation of cobalt (II, III) oxide was consistent with the results of XPS analysis, in which Co (II) and Co (III) were also detected. Other peaks visible in the magnified X-ray diffraction pattern belonged to the Si/Ti/Au substrate. The architecture, structure, and composition of the deposits make them a material with the potential to catalyze the mineralization of organic pollutants via PMS catalytic processes.

### 3.2. Mineralization of TCs via PMS Catalysis

Prior to analyzing the catalytic performance of Ni- and Co–CoNi substrates, the persistence of TCs in the absence of any catalyst and PMS precursor in dark conditions was examined. The time-dependent UV–Vis spectrum of 20 ppm solutions of TCs at a pH of 6.0 and a pH of 8.0 in the absence of any catalyst and dark conditions for 45 d was recorded. In addition, the TOC of fresh pollutant solutions and the solution after 45 d was also determined. The UV–Vis spectra evidenced that the absorbance values slightly decreased at pH = 6.0 and continuously lessened at pH = 8.0 during the period of 45 d. Importantly, the mineralization percentage was also negligible, which affirms its lengthy half-life in aqueous medium and, consequently, the potential threat of the proliferation of new microorganisms resistant to current antibiotics [23].

The effects of the pH and concentration of PMS of the polluted solutions, light irradiation, and the duration of contact with the Ni- and Co–CoNi catalysts on the degradation and mineralization of TCs were analyzed. Prior to determining the catalytic performance of PMS on the mineralization of TCs, the adsorption of the pollutant was investigated (Table S1). The adsorption–desorption equilibrium was achieved within 10 min of contact with the solution of TCs, while the absorbance measurements remained constant. As shown in Table S1, TC adsorption values were virtually constant regardless of the chemical nature of the surfaces of the catalysts and their different morphologies and, in all cases, were approximately 10% of the TCs. Because the TC molecules were neutrally charged at a pH of 6.0 and negatively charged at a pH of 8.0, the molecule should have been more or less attracted to the alloy depending on the surface charge [41]. Although no clear trend was observed, at a pH of 8.0 the adsorption was lower than at a pH of 6.0. At the same time, when the degradation of the antibiotics in the absence of any catalyst and at different concentrations of PMS during a 60-min period was examined, we found that, as the concentration of PMS increased, the degradation of TCs increased as well, for degradation efficiencies of less than 1% (i.e., 0.1 mM PMS), ~1% (i.e., 0.2 mM PMS), and ~2% (i.e., 0.3 mM PMS). Those results demonstrate the need to use catalysts to facilitate the formation of radical species capable of oxidizing TCs and/or other pollutants.

**Figure 4:**
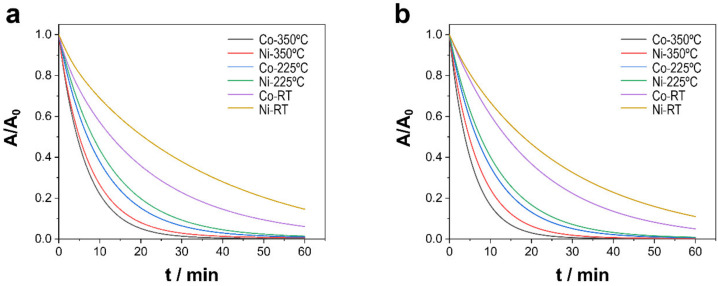
Catalytic degradation of TCs using different Ni–CoNi and Co–CoNi deposits at (**a**) pH 6.0 and (**b**) pH 8.0. Experimental conditions: 20 ppm of TCs [PMS]_0_ = 0.3 mM, T = 20 °C, and dark conditions.

To analyze the capacity of each material to decontaminate water, the degradation of TCs after placing the catalysts and solution in contact in the dark for 30 min in the absence of PMS was examined. After 30 min, 0.1 mL of a PMS solution was added to achieve a concentration of 0.3 mM PMS in the solution. As shown in Figure 4, at time 0, when 0.1 mL of PMS was added into the reactor, how the degradation of TCs was initiated could be fully observed, given an abrupt drop in the concentration of pollutants for all types of catalysts and conditions studied. The first parameters analyzed were the effect of the annealing treatment applied to the sample and the alloy composition (i.e., the chemical nature, crystal structure, and morphology of catalysts), along with the effect of the pH of the media, to examine two pH levels representative of the range of pH levels where most wastewater is found. Although morphological variation indeed influenced catalytic activity, the elemental composition and oxidation state of the surface of the catalysts, as well as the physicochemical properties, determined the activation of PMS and, in turn, its performance in degradation and mineralization. As shown in Figure 4a,b, independent of pH, the degradation of the pollutants followed the same trend. Samples subjected to treatment at 350 °C presented faster degradation than samples treated at 225 °C and, by a greater extent, the as-prepared samples, such that, after 30 min, degradation was nearly complete. Therefore, treatment at a high temperature seems to facilitate the activation of PMS and, consequently, the removal of TCs. The annealing treatment stimulated the oxidation of cobalt and nickel in the substrate and the formation of oxides on the surface, in which the reversible redox cycles of Co^2+^–Co^3+^ and Ni^2+^–Ni^3+^, as well as enhancement in the charge transfer properties, contributed to the generation of sulfate and oxygen radicals, which improved the catalytic efficiency of PMS activation [19,20]. In addition to the annealing treatment, the alloy composition is fundamental to explaining catalytic performance. Indeed, Co–CoNi deposits showed a higher degradation efficiency than Ni–CoNi catalysts independent of pH. Last, pH was another determining factor of the kinetics of the degradation reaction. In particular, at a pH of 8.0, the process was faster than in slightly acidic conditions (i.e., pH = 6.0), which maintained the established order showing that samples treated at 350 °C degraded the quickest, followed by those treated at 225 °C and, in turn, the untreated samples. Because the reactivity of pollutants increases with an increasing degree of deprotonation, at a pH of 8.0, TCs are present as anions and thus have greater reactivity than TCs present as neutral molecules at a pH of 6.0. The calculated values (Table 2) of the pseudo-first-order rate kinetic constants, with the assumptions of the Langmuir–Hinshelwood kinetics model applied, show that the degradation of TCs at a pH of 8.0 increased from 0.045 min^−1^ to 0.1469 min^−1^ for Ni–CoNi (RT) and Ni–CoNi (350 °C), respectively, and from 0.050 to 0.173 min^−1^ for Co–CoNi (RT) and Co–CoNi (350 °C), respectively. Moreover, the kinetic constants for catalysts annealed at 350 °C multiplied in value by more than threefold in relation to the non-annealed catalysts. The Co samples also exhibited greater degradation in light with higher rate constants, and experiments conducted at a pH of 8.0 were the most effective. The mineralization of TCs was also evaluated in light of the TOC measurements (Table 2), the results of which confirm even more strongly what was previously concluded: that Co–CoNi at a pH of 8.0 activated PMS the best and consequently demonstrated the greatest degradation. Even so, it is important to highlight the strong performance of all of the catalysts, for after 1 h, the mineralization achieved by Ni–CoNi and Co–CoNi ranged between 85% and 94% for deposits treated 2 h at 225 °C and between 96% and 99.9% for deposits annealed 2 h at 350 °C

The Co–CoNi (350 °C) deposits exhibited excellent catalytic activity, equal or even superior to state-of-the-art catalysts used to degrade and mineralize TCs via PMS catalysis, including petal-like hierarchical Co_3_O_4_- or N-doped porous carbon [42], partly carbonized Fe_3_O_4_@PANI-p [43], N-C codoped Fe_2_O_3_ [44], Co/N codoped biochar [45], goethite-MoS_2_ hybrid [46], porous CuFe_2_O_4_ [47], and biochar supported-Co_3_O_4_ [48], among others.

#### 3.2.1. Effect of PMS Concentration

**Figure 5:**
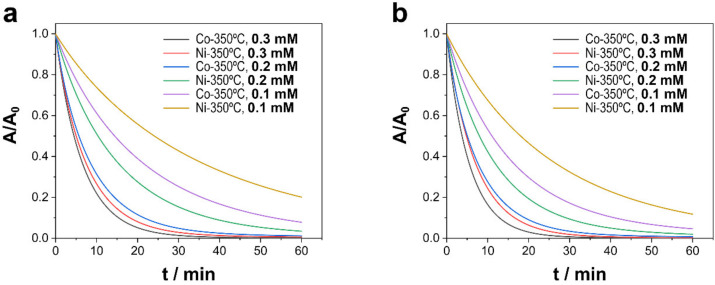
Catalytic degradation of TCs as a function of the concentration of PMS using different Ni–CoNi and Co–CoNi deposits annealed at 350 °C at (**a**) a pH of 6.0 and (**b**) a pH of 8.0. Experimental conditions: 20 ppm of TCs [PMS]_0_ = 0.1 to 0.3 mM, T = 20 °C, and dark conditions.

As shown in Figure 5, the concentration of PMS was a determining factor in the degradation of TCs. We observed that as the concentration of PMS decreased, the degradation rate decreased as well. Figure 5 shows only the degradation of deposits subjected to annealing at 350 °C, whereas in deposits in which the presence of Ni (0) was not insignificant, especially in Ni–CoNi (RT) deposits, degradation fell rapidly as the concentration of PMS decreased. The presence of Ni(0) seemed to force the concentration of PMS to increase as a result of the competitive reaction between Ni (0) and PMS in which no radicals were formed (Equation (3)) [19,49].

(3) $$HSO_{5}^{-}+Ni\to Ni^{2+}+SO_{4}^{2-}+OH^{-}$$

Table 3 summarizes the apparent kinetic constants of degradation as a function of the concentration of PMS. Considering deposits in which Ni (0) content was insignificant, the kinetics of degradation increased up to a concentration of PMS of 0.3 mM. At higher concentrations, the kinetics of degradation worsened as a result of the parasite self-scavenging reaction (Equation (4)) and the reaction of PMS with sulfate radicals (Equation (5)) [49,50]:

(4) $$HSO_{5}^{-}+SO_{4}^{●-}\to SO_{5}^{●-}+SO_{4}^{2-}+H^{+}$$

(5) $$SO_{4}^{●-}+SO_{4}^{●-}\to SO_{8}^{2-}$$

Based on our results, the concentration of PMS is a critical factor due to several parasitic reactions between sulfate radicals as well as between sulfate radicals and PMS that occur amid high concentrations of PMS. Other secondary reactions may also exert an influence, as in the case of Ni (0), in which electron transfer from zerovalent nickel to PMS occurs without generating free radicals. However, the self-scavenging reaction is more relevant when the adsorption of pollutants is lower and when the concentration of pollutants is smaller.

#### 3.2.2. Effect of Light Irradiation

The performance of the catalysts was also investigated under visible light irradiation. In general, PMS can be photolytically activated via the following mechanisms based on the breaking of the O–O bond (Equation (6)) or on the reaction of PMS with the photogenerated radicals from water photolysis (Equation (7)) [27,50,51]:

(6) $$HSO_{5}^{-}+hv\to SO_{4}^{●-}+HO^{●}$$

(7) $$HSO_{5}^{-}+H^{●}\to SO_{4}^{●-}+H_{2}O$$

**Figure 6:**
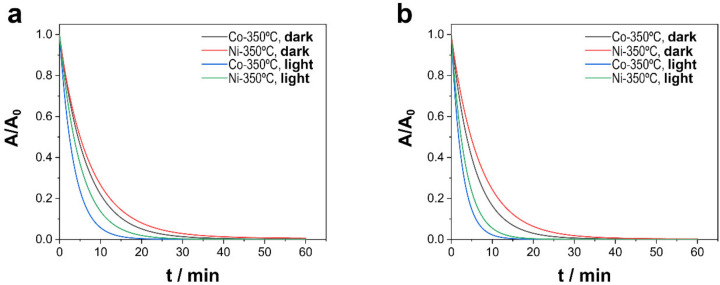
Catalytic degradation of TCs as a function of the light irradiation of Ni–CoNi and Co–CoNi deposits thermal treated during 2 h at 350 °C at (**a**) pH 6.0 and (**b**) pH 8.0. Experimental conditions: 20 ppm of TCs, [PMS]_0_ = 0.3 mM, T = 20 °C, and in dark conditions or under visible light irradiation (i.e., 5 mW cm^−2^).

As shown in Figure 6, the degradation process was much faster than those previously reported, for after 5 min a strong decrease in TCs was observed in presence of visible light irradiation. That outcome was confirmed by apparent values of kinetic constants (Table 4), which were approximately two times greater than ones obtained in dark conditions. TOC measurements also confirmed that the light irradiation of the catalytic system was a determining factor of the activation of PMS and the rapid degradation of TCs, resulting in a nearly 100% degree of mineralization for both catalysts in pH 6.0 and 8.0 conditions.

Irradiation with visible light had a noticeable effect in all cases, while in the absence of any catalyst, the effect of visible light was negligible. The material and light irradiation showed some synergistic effect, especially in Co-rich CoNi deposits, where the rate constant was multiplied by 2.2 and by 1.8 in the case of Ni-rich CoNi deposits. Surprisingly, the rate of degradation increased with the same ratio regardless of the pH and depended only on the chemical nature of the material. Faster degradation was observed at a pH of 8.0, consistent with previous experiments. This synergy can be attributed to the photocatalytic properties of Ni and Co oxides formed during the annealing treatment. However, the photocatalytic activity exerted to degrade and mineralize TCs in the absence of PMS was practically negligible, which reinforces the existence of some synergistic effect between the elements.

#### 3.2.3. Effect of Contact Duration

**Figure 7:**
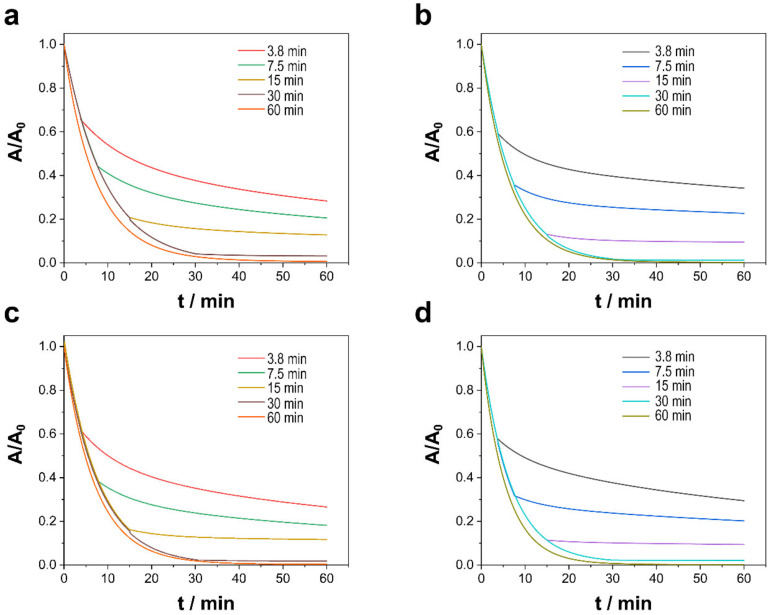
Catalytic degradation of TCs as a function of the catalyst contact duration of (**a**,**c**) Ni–CoNi and (**b**,**d**) Co–CoNi deposits at (**a**,**b**) pH 6.0 and (**c**,**d**) pH 8.0. Experimental conditions: 20 ppm of TCs, [PMS]_0_ = 0.3 mM, T = 20 °C, in dark conditions.

The duration of the catalysts’ contact with the TCs and PMS solution was studied to ascertain its influence on PMS activation. That factor was examined at durations of 3.8, 7.5, 15, 30, and 60 min, after which the annealed alloy was removed, and the degradation reaction was allowed to continue for a total time of 60 min. As shown in Figure 7, the duration of the catalysts’ contact with the polluted solution plays a pivotal role with respect to factors such as pH and the type of catalyst, for the degradation rate decreased drastically when the alloy was removed, but the reaction surprisingly continued even when the catalyst was absent. Despite being heterogeneous catalytic processes and thus processes of surface degradation and mineralization, degradation and, to a lesser extent, mineralization (Table 5) presented a memory effect that allowed degradation to continue but at a slightly lower speed as occurred in a free-radical reaction in which the initiation step is minimized. Moreover, especially in the case of long durations of contact, the longer that the catalysts were in contact with the contaminated solution, the greater the degradation and mineralization once the catalysts were removed. Although unable to detect Ni (II) or Co (II) in the medium, traces of metallic ions can also prompt PMS activation and consequently promote degradation and mineralization. Although the addition of small concentrations of Ni (II) and Co (II) to the reaction medium cannot explain those results, their addition is relevant because it can affect the design of reactors and reduce the cost of decontamination. For that reason, further research is needed to illuminate the phenomenon.

#### 3.2.4. Identification of Free Radicals

To investigate the effect of free radicals on the degradation of TCs formed during the activation of PMS with Co–CoNi (350 °C), we used MeOH TBA as scavengers to clarify the role of hydroxyl and sulfate radicals. In general, MeOH can rapidly quench hydroxyl and sulfate radicals, whereas TBA can quench only hydroxyl radicals because the kinetics with hydroxyl is faster than that of sulfate radicals [52,53,54]. As shown in Table 6, in dark conditions, adding MeOH (50 mM) significantly reduced degradation and achieved rates of only 43% (pH = 6.0) and 48% (pH = 8.0). At the same time, adding TBA (50 mM) produced a far less-pronounced reduction in degradation, with rates of 78% (pH = 6.0) and 82% (pH = 8.0) after 60 min. Under visible light irradiation, the difference in the reduced degradation of both scavengers was more pronounced, which suggests that hydroxyl radicals play a more predominant role under visible light irradiation than in dark conditions. Therefore, even though sulfate and hydroxyl radicals are formed during the activation of PMS using Co–CoNi (350 °C), sulfate radicals seem to have a predominant effect on the degradation process. In addition, under visible light irradiation, the role of hydroxyl radicals is more important than that of sulfate radicals, which nevertheless play an essential role. In short, irradiation with visible light potentiates the formation of hydroxyl radicals during the activation of PMS.

#### 3.2.5. Stability and Reusability of Co-CoNi (350 °C)

**Figure 8:**
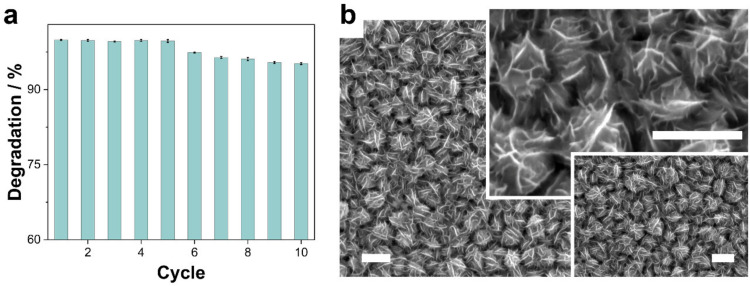
(**a**) Degradation ratio (60 min) of TC using Co–CoNi annealed at 350 °C catalyst for 10 reusability cycles; (**b**) FE-SEM micrographs Co–CoNi annealed at 350 °C after use for 10 reusability cycles. Scale bar: 1 μm.

The stability and reusability of Co–CoNi annealed at 350 °C and only at a pH of 8.0 were evaluated in 10 consecutive cycles for their catalytic activity, morphological integrity, and surface chemical nature by means of XPS analysis (Figure 8). As shown in Figure 8a, the removal efficiency was virtually constant for the first 7 cycles and slightly decreased to 96.4% after 10 cycles. The slight reduction in catalytic performance during the reusability test could be a consequence of the adsorption of intermediate products of the degradation of TCs, catalyst leaching, or changes in the surface morphology and/or chemistry. Cobalt and nickel leaching is negligible; the ICP-OES analysis confirmed that the total concentration of cobalt and nickel ions, considering the 10 solutions treated, was less than 3% of the total amount of cobalt and nickel in the deposits (i.e., ~0.001 mg mL^−1^ of cobalt and ~0.0001 mg mL^−1^ of nickel). The FE-SEM of reused deposits confirmed the robustness of the Co–CoNi deposits annealed at 350 °C, given the lack of any significant changes in the morphology of the material (Figure 8b). In addition, at the milli- and micro-metric level, no damage or detachment of fragments of the film was observed. When the Co–CoNi (350 °C) catalyst, already used 10 times, was cleaned, dried, and again subjected to annealing at 350 °C for 2 h, the catalytic activity recovered, even to levels exceeding 99% degradation of TCs. These results suggest that the degradation level achieved by the Co–CoNi (350 °C) catalyst can be restored.

Last, to probe the stability and reusability of the Co–CoNi (350 °C) catalysts and the activation of PMS, the XPS spectra of Co 2p and Ni 2p after 10 reusability cycles were investigated. Figure S2 shows high-resolution spectra of Co 2p and Ni 2p of the Co–CoNi (350 °C) catalyst used. After 10 catalytic reusability cycles, the Co (II)-to-Co (III) ratio slightly decreased, while the Ni (II)-to-Ni (III) ratio slightly increased, which suggests that the activation of PMS follows the typical mechanism via electron transfer, in which Co–CoNi annealed at 350 °C catalyst forms an active reversible redox cycle of Co^2+^–Co^3+^ and Ni^3+^–Ni^2+^ and generates sulfate and hydroxyl radicals [27].

## 4. Conclusions

In our study, we have demonstrated the synthesis of Ni-rich and Co-rich CoNi-based catalysts employed to activate PMS for use in degrading and mineralizing TCs. Our simple fabrication of catalysts was based on a low-cost electrodeposition process using a green DES as an electrochemical bath that allowed the control and tuning of the morphology, thickness, structure, and chemical nature of the catalysts. Although the oxidation of electrodeposited Ni- and Co-rich CoNi micro-nanostructured films has been studied as a means to promote the formation of cobalt and nickel oxides, which are more effective in activating PMS, oxidized Co-CoNi deposits are more efficient than those rich in nickel (Ni-CoNi). Even so, Ni and Co present synergistic effects that improve the catalytic activity and stability of the material. In addition, oxidized Co-rich CoNi deposits have comparable or even better catalytic activity than state-of-the-art PMS catalysts for antibiotic degradation. Among our results, all of the prepared materials were effective at pH levels ranging from 6.0 to 8.0—that is, the pH levels most often found in polluted waters.

The concentration of PMS can become a critical factor in water treatment, for very small concentrations promote a low rate of sulfate radical formation and therefore little catalytic activation. However, high concentrations of PMS also end up reducing catalytic activity as a result of parasitic reactions, including self-scavenging ones. Meanwhile, the presence of Ni(0) exerts negative effects by consuming PMS without generating sulfate radicals. Considering those influences, the optimal concentration in our study was 0.3 mM of PMS.

Light irradiation seems to be a pivotal factor. Although the effect of visible light cannot be explained, catalytic activity increased significantly in our study when the reaction medium was irradiated. Among other factors, the photolysis of water and PMS contributed to improved catalysis but cannot sufficiently justify the increase in catalytic performance. The irradiation of the material in the absence of PMS also does not explain the kinetics of degradation, meaning that some cooperative effect occurred between the PMS, the light, and the catalyst.

The duration of contact can also affect the design of reactors. Our study has shown that degradation and mineralization continue, albeit with different kinetics, when the catalyst and the solution containing the contaminant are separated. In addition, it seems that the catalytic degradation process is primarily based on the formation of sulfate radicals, though the role of hydroxyl radicals cannot be discounted.

In contribution to future research, our results suggest that PMS activation is based on the typical mechanism of electron transfer, which forms an active reversible redox cycle of Co^2+^–Co^3+^ and Ni^3+^–Ni^2+^ and generates sulfate and hydroxyl radicals. The excellent reusability and stability of oxidized Co-rich CoNi deposits also reinforce the potential of the material for PMS catalysis in the context of water treatment. Those results indicate new directions for the preparation of effective catalysts for environmental applications.

## Figures and Tables

**Table 1:** Electrochemical media, deposition conditions, and elemental composition of prepared deposits.

Electrochemical Media	E/V (vs. Ag|AgCl)	Co/at. %		Ni/at. %	
		EDS	ICP	EDS	ICP
0.14 M NiCl_2_ and 0.03 M CoCl_2_ in a 1 ChCl:2 urea (molar ratio) deep eutectic solvent	−0.95	16.2	15.4	83.8	84.6
0.03 M NiCl_2_ and 0.14 M CoCl_2_ in a 1 ChCl:2 urea (molar ratio) deep eutectic solvent	−1.05	84.0	84.2	16.0	15.8

**Table 2:** Kinetic constants of the degradation and mineralization of TCs using the Ni–CoNi and Co–CoNi deposits subjected to different thermal treatments.

	pH = 6.0		pH = 8.0	
	k/min^−1^	Mineralization/%	k/min^−1^	Mineralization/%
Ni-CoNi (RT)	0.031	65.9 ± 0.2	0.045	70.4 ± 0.3
Ni-CoNi (225 °C)	0.077	85.9 ± 0.1	0.088	89.8 ± 0.2
Ni-CoNi (350 °C)	0.105	96.0 ± 0.2	0.147	97.8 ± 0.3
CoNi (RT)	0.037	78.4 ± 0.4	0.050	81.0 ± 0.4
Co-CoNi (225 °C)	0.086	90.1 ± 0.3	0.096	94.4 ± 0.2
Co-CoNi (350 °C)	0.132	99.4 ± 0.3	0.173	99.9 ± 0.1

**Table 3:** Kinetic constants of the degradation of TCs as a function of the concentration of PMS.

PMS Concentration/mM	k/min^−1^ Ni-CoNi (350 °C)		k/min^−1^ Co-CoNi (350 °C)	
	pH = 6.0	pH = 8.0	pH = 6.0	pH = 8.0
0.1	0.027	0.044	0.036	0.055
0.2	0.060	0.103	0.075	0.115
0.3	0.105	0.147	0.132	0.173
0.4	0.077	0.109	0.081	0.122
1.0	0.016	0.026	0.023	0.034

**Table 4:** Kinetic constants of the degradation of TCs in dark conditions and under visible light irradiation (i.e., 5 mW cm^−2^).

Conditions	k/min^−1^ Ni-CoNi (350 °C)		k/min^−1^ Co-CoNi (350 °C)	
	pH = 6.0	pH = 8.0	pH = 6.0	pH = 8.0
Dark conditions	0.105	0.147	0.132	0.173
Visible light irradiation	0.199	0.292	0.288	0.388

**Table 5:** Mineralization of TCs as a function of the duration of contact with the catalysts.

Contact Time/min	Mineralization/% Ni-CoNi (350 °C)		Mineralization/% Co-CoNi (350 °C)	
	pH = 6.0	pH = 8.0	pH = 6.0	pH = 8.0
3.8	43 ± 2	46 ± 3	42 ± 1	51 ± 2
7.5	56 ± 2	59 ± 1	61 ± 1	65 ± 2
15	73 ± 3	78 ± 2	69 ± 2	75 ± 3
30	82 ± 2	86 ± 1	83 ± 2	89 ± 1
60	96.0 ± 0.2	97.8 ± 0.3	99.4 ± 0.3	99.9 ± 0.1

**Table 6:** Degradation of TC in dark conditions and under visible light irradiation (i.e., 5 mW cm^−2^) in the presence and absence of scavengers.

Scavenger	Degradation/%			
	Dark Conditions		Visible Light Irradiation	
	pH = 6.0	pH = 8.0	pH = 6.0	pH = 8.0
No scavenger	>99	>99	>99	~100
MeOH	43.2 ± 0.2	48.4 ± 0.1	>99	>99
TBA	78.1 ± 0.3	82.6 ± 0.2	>99	>99

## Data Availability

Data available upon request.

## Supplementary Materials

The following supporting information can be downloaded at: https://www.mdpi.com/article/10.3390/nano13050790/s1, Table S1: Adsorption values of TC respect to the initial concentration of TC in the fresh solution after 30 min in dark and in absence of PMS when the adsorption-desorption equilibrium was reached; Figure S1: Thermal treatment process at (**a**) 225 °C and (**b**) 350 °C; Figure S2: XPS spectra of the reused Co-CoNi deposits of (**a**) Co 2p and (**b**) Ni 2p.
