# Supplementary material for: Enhanced Activation of Peroxymonosulfate for Tetracycline Degradation Using CoNi-Based Electrodeposited Films
Source: Nanomaterials (Basel). 2023 Feb 21;13(5):790. doi: 10.3390/nano13050790 (PMC10005342; doi:10.3390/nano13050790)
Supplement: Supplementary file 1 [file nanomaterials-13-00790-s001.zip › nanomaterials-2241060-supplementary.pdf]

nanomaterials

Supplementary Materials

Enhanced Activation of Peroxymonosulfate for Tetracycline Degradation Using CoNi-Based Electrodeposited Films

Elvira Gómez [1,2,*], Arnau Fons [1], Roberto Cestaro [3] and Albert Serrà [1,2,*]

[1] Grup d'Electrodeposició de Capes Primes i Nanoestructures (GE-CPN), Departament de Ciència de Materials i Química Física, Universitat de Barcelona, Martí i Franquès, 1, 08028 Barcelona, Catalonia, Spain
[2] Institute of Nanoscience and Nanotechnology (IN²UB), Universitat de Barcelona, 08028 Barcelona, Catalonia, Spain
[3] Empa Swiss Federal Laboratories for Materials Science and Technology, Laboratory for Joining Technologies and Corrosion, CH-8600 Dübendorf, Switzerland
* Correspondence: e.gomez@ub.edu (E.G.); a.serra@ub.edu (A.S.)

1. Adsorption-Desorption Equilibrium

Table S1. Adsorption values of TC respect to the initial concentration of TC in the fresh solution after 30 min in dark and in absence of PMS when the adsorption-desorption equilibrium was reached.

	**pH = 6.0**	**pH = 8.0**
Ni-CoNi RT	11.2 ± 0.2	10.4 ± 0.2
Ni-CoNi 225 °C	10.2 ± 0.1	10.3 ± 0.3
Ni-CoNi 350 °C	11.6 ± 0.2	10.6 ± 0.2
Co-CoNi RT	10.2 ± 0.2	10.5 ± 0.2
Co-CoNi 225 °C	10.6 ± 0.3	10.5 ± 0.1
Co-CoNi 350 °C	9.6 ± 0.3	9.5 ± 0.2

2. Thermal Treatment

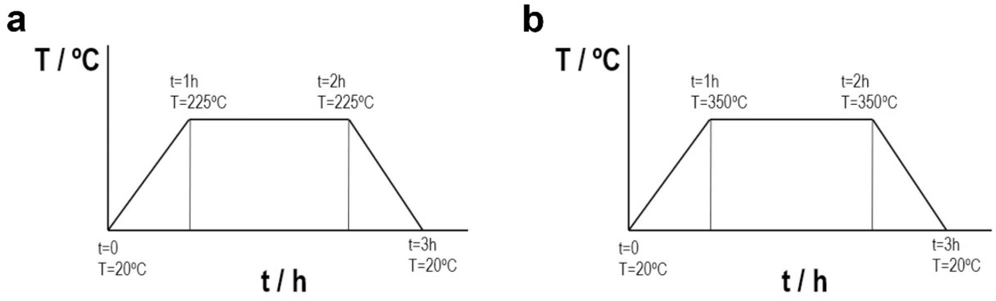

Figure S1. Thermal treatment process at (**a**) 225 °C and (**b**) 350 °C.

3. XPS

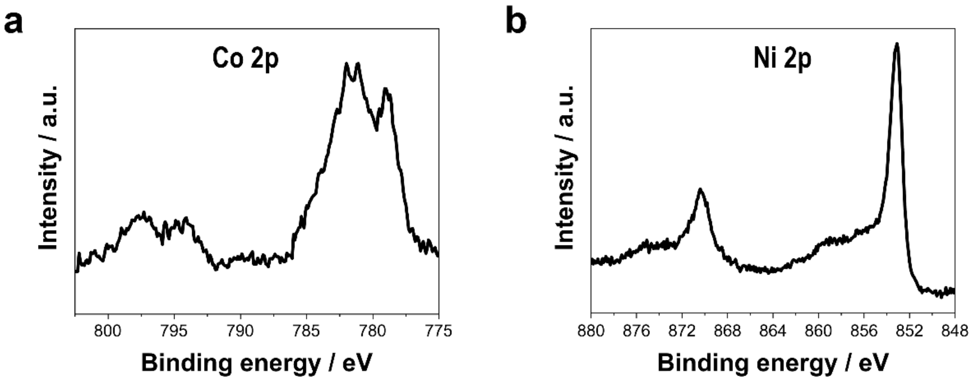

Figure S2. XPS spectra of the reused Co-CoNi deposits of (**a**) Co 2p and (**b**) Ni 2p.
